# Design, Synthesis, and Biological Evaluation of a Novel [^18^F]AlF-H_3_RESCA-FAPI Radiotracer Targeting Fibroblast Activation Protein

**DOI:** 10.3390/ph18020277

**Published:** 2025-02-19

**Authors:** Qingyu Zhang, Zhoumi Hu, Haitao Zhao, Fuqiang Du, Chun Lv, Tukang Peng, Yukai Zhang, Bowu Zhang, Jianjun Liu, Cheng Wang

**Affiliations:** 1 Department of Nuclear Medicine, Renji Hospital, School of Medicine, Shanghai Jiao Tong University, Shanghai 200127, China; 1000528893@smail.shnu.edu.cn (Q.Z.); huzhoumi@renji.com (Z.H.); zht870929@163.com (H.Z.); dfq999@126.com (F.D.); lvchun@renji.com (C.L.); pengtukang@sjtu.edu.cn (T.P.); 18834697969@163.com (Y.Z.); 2 MOE Key Lab of Resource Chemistry, Joint International Research Laboratory of Resource Chemistry of Ministry of Education, Shanghai Key Laboratory of Rare Earth Functional Materials, and Shanghai Frontiers Science Centre of Biomimetic Catalysis, Shanghai Normal University, Shanghai 200234, China; zhangbowu@shnu.edu.cn

**Keywords:** H_3_RESCA chelator, fibroblast activation protein inhibitor, fluorine-18, positron emission tomography

## Abstract

**Background:** Cancer-associated fibroblasts (CAFs) are key contributors to the tumorigenic process, with fibroblast activation protein (FAP) overexpressed on CAFs in numerous epithelial carcinomas. FAP represents a promising target for tumor imaging and therapy. We aimed to develop a novel [^18^F]AlF-H_3_RESCA-FAPI radiotracer with a high labeling yield at room temperature for positron emission tomography (PET) imaging of FAP-expressing tumors. **Methods**: The H_3_RESCA-FAPI chelator was synthesized and radiolabeled with [^18^F]AlF. Its radiotracer binding affinity to FAP was assessed using surface plasmon resonance (SPR). Its in vitro stability, plasma clearance, and biodistribution were evaluated. PET imaging was performed in U87MG tumor-bearing mice, with a blocking study to assess tracer specificity. **Results**: The [^18^F]AlF-H_3_RESCA-FAPI radiotracer demonstrated a high binding affinity to FAP (K_D_ < 10.09 pM) and favorable radiochemical yields (92.4 ± 2.4%) with >95% radiochemical purity. In vitro and in vivo studies showed good stability and rapid clearance from non-target tissues. PET imaging revealed specific tumor uptake, which was significantly reduced by co-injection with unlabeled DOTA-FAPI-04. **Conclusions**: [^18^F]AlF-H_3_RESCA-FAPI is a promising radiotracer for PET imaging of FAP-expressing tumors. Further optimization of its pharmacokinetics could make it a potential candidate for clinical translation.

## 1. Introduction

The tumor microenvironment (TME), includes fibroblasts, myeloid-derived suppressor cells (MDSCs), macrophages, lymphocytes, the extracellular matrix (ECM), and intertwined blood vessels constructed by endothelial cells and pericytes [1]. These stromal cells tightly interact with cancer cells, creating a supportive environment for tumor growth. Among these, cancer-associated fibroblasts (CAFs) play a crucial role in tumor progression by contributing to processes such as tumorigenesis, neoangiogenesis, metastasis, immunosuppression, and drug resistance [2,3]. Fibroblast activation protein (FAP), a type II transmembrane serine protease belonging to the dipeptidyl peptidase 4 family with both dipeptidyl peptidase and endopeptidase activity [4], is overexpressed on CAFs in more than 90% of epithelial carcinomas [5,6,7,8].

**Figure 1:**
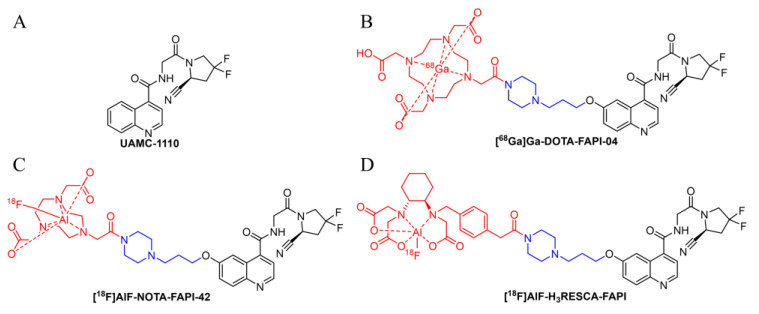
The structures of UAMC-1110 (**A**), [^68^Ga]Ga-DOTA-FAPI-04 (**B**), [^18^F]AlF-NOTA-FAPI-42 (**C**), and [^18^F]AlF-H_3_RESCA-FAPI (**D**). The blue part is the linker, the red part is the chelator group, and the black part is FAPI pharmacophore.

Therefore, FAP is considered a promising target for both tumor imaging and therapy. In 2014, UAMC-1110 (Figure 1A) was identified as the most potent and selective small molecule inhibitor of FAP [9,10]. Since then, some classic UAMC-1110-based PET tracers have been developed [11,12,13], such as [^68^Ga]Ga-DOTA-FAPI-04 (Figure 1B) and [^18^F]AlF-NOTA-FAPI-42 (Figure 1C).

Initially, [^68^Ga]Ga-DOTA-FAPI-04 garnered the majority of attention within the FAPI diagnostics field [14]; however, current research is also focusing on ^18^F-labeled FAPIs due to the excellent spatial resolution theoretically obtained by ^18^F-PET imaging [15] and the relatively long half-life of ^18^F (t_1/2_ = 109.8 min, compared with t_1/2_ (^68^Ga) = 67.7 min). Furthermore, the limited availability of ^68^Ga, which must be produced in relatively small batches through ^68^Ge/^68^Ga generators, contributes to the growing interest in ^18^F-labeled FAPIs. In contrast, ^18^F can be mass produced in cyclotrons or transported to PET imaging centers, which lack radionuclide production capacity [16,17].

The labeling techniques for ^18^F can be categorized into two groups: nucleophilic ^18^F-derivatives generated by various substitution reactions [15,18,19] and the utilization of aluminum fluoride ([^18^F]AlF) for radiolabeling via coordination chemistry [20]. The method of using [^18^F]AlF labeling has been expanding, and a series of different chelator groups have achieved great success, such as NOTA, NODA, NODAGA, 2-AMPTA, and DTPA; most of these have certain requirements for equipment [21].

Recently, H_3_RESCA has been widely used in the ^18^F labeling of antibodies because of its mild reaction conditions and straightforward labeling process [22,23], which has piqued our interest. Our research aims to utilize H_3_RESCA for the labeling of small molecules with ^18^F, using FAPI as an example to broaden the potential applications of FAPI in nuclear medicine (Figure 1D).

## 2. Results

### 2.1. Docking Simulations

**Figure 2:**
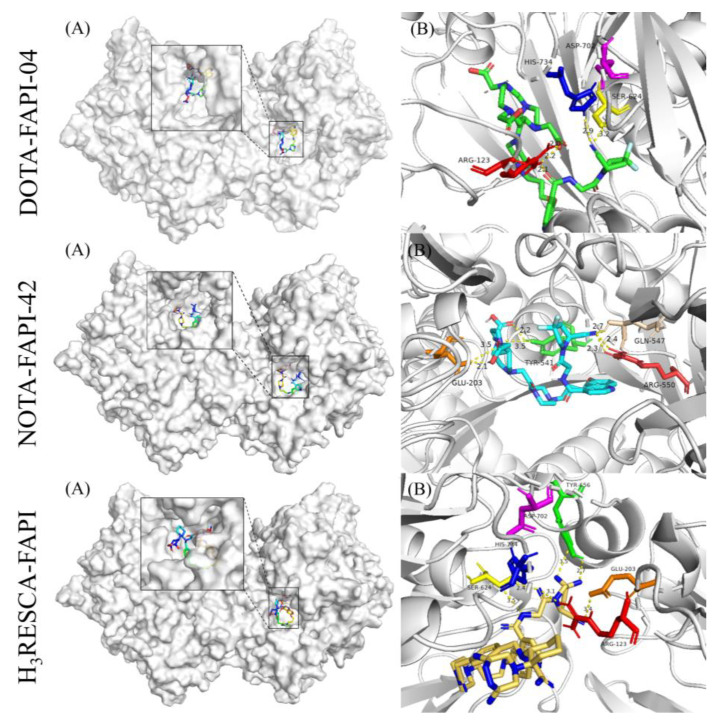
Molecular modeling: (**A**) FAP ligand binding cavity of FAPIs; and (**B**) Interaction diagram between amino acid residues of FAPIs and FAP in ligand binding pocket. The amino acid residues were color-coded as follows: red for ARG-(123 or 550), orange for GLU-203, yellow for SER-624, green for TYR-(541 or 656), blue for HIS-734, purple for ASP-702, and flesh color for GLN-547.

Molecular docking simulations were conducted to elucidate the interactions between FAPI-based ligands and the FAP enzyme, with the results depicted in Figure 2 demonstrating that the introduction of this innovative chelating agent group enhanced the interaction. Specifically, H_3_RESCA-FAPI exhibited a wider range of interactions with amino acid residues, including His^734^, Ser^624^, Arg^123^, Tyr^656^, and Glu^203^, compared to DOTA-FAPI-04, which primarily interacted with Ser^624^, His^734^, and Arg^123^, and NOTA-FAPI-42, which interacted with Glu^203^, Tyr^541^, Gln^547^, and Arg^550^. These interactions were further characterized by the presence of potential hydrogen bonds, indicated by the yellow dotted lines in the figure. Detailed scoring of these interactions is provided in Table 1.

### 2.2. ADMET Profiling Study

In the preliminary stages of computer-aided drug design (CADD), the absorption, distribution, metabolism, excretion, and toxicity (ADMET) profiles of chemical compounds are acknowledged as critical factors. The pharmacokinetic properties and drug-likeness metrics for these compounds are detailed in Tables S1, S2, and S3, respectively.

A pharmacokinetic analysis indicates that H_3_RESCA-FAPI possesses a higher intestinal absorption (HIA) profile compared to DOTA-FAPI-04, which exhibits lower absorption rates. Both ligands demonstrated a limited ability to cross the blood–brain barrier. The human colon epithelial cancer cell line Caco-2 serves as a surrogate for studying drug intestinal absorption in humans. Permeability studies using the Caco-2 model revealed no significant difference between the two ligands in terms of membrane permeation. Additionally, drug-likeness was assessed based on the Lipinski rule, Pfizer rule, GSK rule, and golden triangle rule. Both compounds adhered to the Pfizer rule; however, they did not comply with the Lipinski, GSK, and golden triangle criteria. For radioactive diagnostic agents, the administered doses are typically very low, thus mitigating concerns regarding the chemical toxicity of such drugs. In summary, the simulation experiments indicate that the two inhibitors are largely comparable, with the primary differences being their lipid solubility and intestinal uptake. The variance in lipid solubility may underlie the observed differences in intestinal absorption.

### 2.3. Binding Affinity

**Figure 3:**
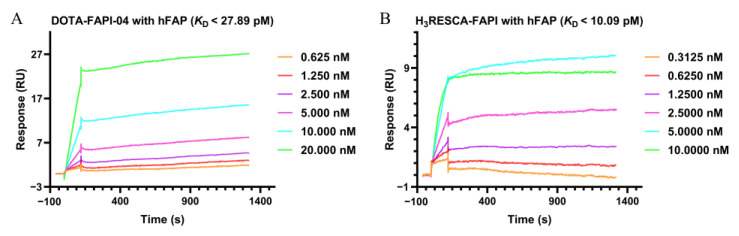
(**A**) DOTA-FAPI-04 and (**B**) H_3_RESCA-FAPI reactive curves with recombinant human FAP protein.

SPR was utilized to evaluate the interaction between DOTA-FAPI-04 and H_3_RESCA-FAPI with recombinant human FAP protein (Figure 3). The equilibrium dissociation constant (K_D_) value of the two inhibitors binding to human FAP proteins exhibited a robust affinity, being in the picomole (pM) range. Specifically, the K_D_ value of DOTA-FAPI-04 is less than 27.89 pM, while the K_D_ value of H_3_RESCA-FAPI is less than 10.09 pM. This shows that the affinity of H_3_RESCA-FAPI for FAP protein is slightly higher than that of DOTA-FAPI-04, which is consistent with the results of the molecular docking simulation.

The molecular docking simulations and ADMET profiling study established the structural feasibility of H_3_RESCA-FAPI, predicting its interactions with FAP and its pharmacokinetic properties. The high binding affinity of H_3_RESCA-FAPI to FAP, as determined by SPR, is consistent with the docking results and underscores the potential of this tracer for the specific targeting of FAP-expressing tumors.

### 2.4. Chemical and Radiochemical Synthesis

**Scheme 1:**
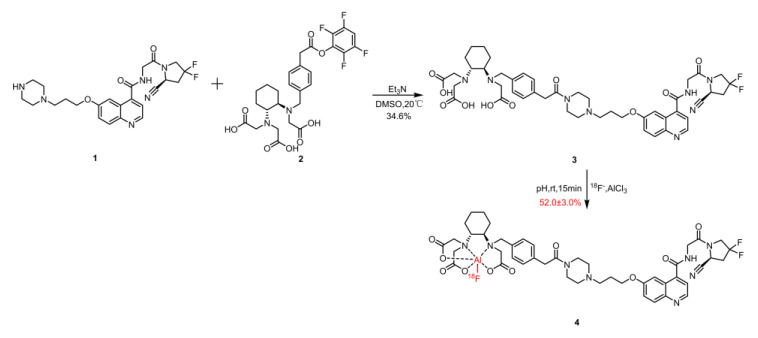
Synthesis of [^18^F]AlF-H_3_RESCA-FAPI.

H_3_RESCA-FAPI (compound **3**) was synthesized (Scheme 1), and its molecular weight was identified by LC-MS (Figure S1).

**Figure 4:**
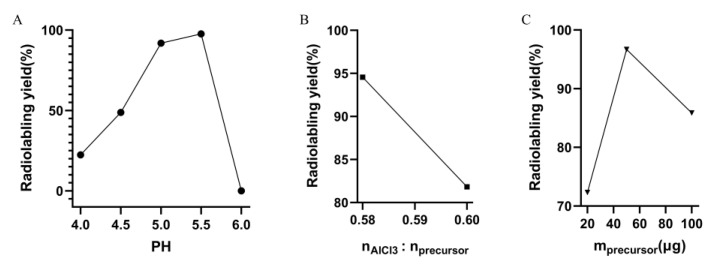
Labeling yield of [^18^F]AlF-H_3_RESCA-FAPI under different reaction conditions. Labeling yield under different pH values (4.0, 4.5, 5.0, 5.5, and 6.0) of buffer system (**A**); The ligand mass was fixed (20 μg) with different AlCl_3_–ligand ratios (0.58 and 0.60) (**B**); The ligand ratio of AlCl_3_ is fixed (the molar ratio of substances is 0.58), and the labeling yield is different under different ligand masses (20 μg, 50 μg, and 100 μg) (**C**).

The ^18^F-labeled FAP tracer was generated via the formation of complexes of Al^18^F in a two-step reaction. According to the lateral comparison, the reaction conditions with the highest labeling yield were as follows: the buffer pH = 5.0, the mole ratio of AlCl_3_ to the precursor was 0.58, and the weight of H_3_RESCA-FAPI was 50 μg (Figure 4 and Table S4). A highly reproducible radiolabeling yield of 95% was obtained under the optimal conditions.

**Figure 5:**
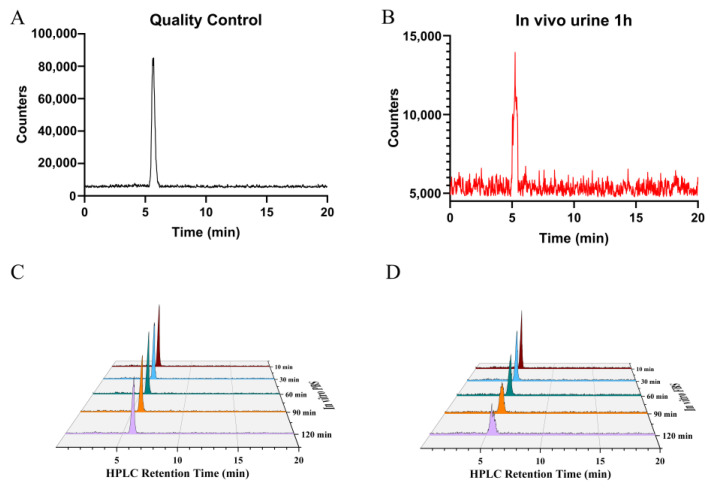
Representative HPLC graphs of quality control of [^18^F]AlF-H_3_RESCA-FAPI. (**A**) In vivo metabolism study of [^18^F]AlF-H_3_RESCA-FAPI at 1 h p.i. (**B**) and its stability at 37 °C for 2 h in PBS (**C**) and FBS (**D**).

Under the optimum labeling conditions, the total time needed for radiosynthesis was approximately 20 min. The non-decay-corrected radiochemistry yield (RCY) of [^18^F]AlF-H_3_RESCA-FAPI was 86.1 ± 4.5%, while the decay-corrected RCY was 92.4 ± 2.4% (*n* = 6). The radiochemical purity (RCP) of [^18^F]AlF-H_3_RESCA-FAPI was over 95% with molar activities of more than 14.5 GBq/μmol (*n* = 6) according to radioactivity measurements (Figure 5A, Figures S2 and S3). The labeling yield is not only related to the three factors screened (the pH of the buffer, the ratio of AlCl_3_ to the precursor, and the dosage of the precursor), but is also influenced by other factors (the room temperature and the concentration of the reaction solution), resulting in significant variability in labeling yields under identical conditions. During the optimization process, it was observed that the reaction volume significantly influenced the labeling yield. Specifically, increasing the reaction volume from approximately 220 μL to 310 μL while maintaining constant conditions (ligand mass = 20 μg, AlCl_3_/precursor molar ratio = 0.58) resulted in a modest increase in radiolabeling yield from 91.88% to 94.56% (Figure 4A,B). This observation underscores the importance of meticulously controlling the reaction volume to ensure reproducibility.

Additionally, while the primary objective of our study was to develop a radiotracer capable of achieving a high labeling efficiency without stringent temperature regulation, it is acknowledged that minor fluctuations in ambient temperature could potentially affect the reaction efficiency. All reactions were conducted at ambient room temperature on the day of the experiment, without active temperature control or monitoring. Although the specific impact of temperature variations was not investigated in this study, it is recognized that this factor may contribute to the observed variability in labeling yields under otherwise identical conditions.

### 2.5. Octanol–Water Partition Coefficient and Stability Assay

The octanol–water partition coefficient, expressed as logD_7.4_, for the radiopharmaceuticals [^68^Ga]Ga-DOTA-FAPI-04 and [^18^F]AlF-H_3_RESCA-FAPI were calculated to be −3.53 ± 0.05 and −2.47 ± 0.16, respectively. This comparative analysis suggests that [^18^F]AlF-H_3_RESCA-FAPI possesses a reduced hydrophilicity in comparison to [^68^Ga]Ga-DOTA-FAPI-04. Given the constraints inherent in ADMETLab 3.0 regarding the processing of SMILES files that include metal complexes, the distribution coefficients of the respective uncomplexed precursors served as a benchmark for assessing the hydrophilicity of these radiopharmaceutical agents (the logP values of DOTA-FAPI-04 and H_3_RESCA-FAPI are −1.982 and −0.639, respectively). The observed hydrophilicity trends corroborate the simulation results obtained for the labeled precursors during the ADMET prediction analyses. It demonstrated that [^18^F]AlF-H_3_RESCA-FAPI has a reduced hydrophilicity compared to [^68^Ga]Ga-DOTA-FAPI-04, which may influence its biodistribution and pharmacokinetics. Furthermore, the stability assessment of [^18^F]AlF-H_3_RESCA-FAPI in phosphate-buffered saline (PBS, pH = 7.4), fetal bovine serum (FBS), and mouse urine—presented in Figure 5, underscores the tracer’s robust stability in both in vitro and in vivo conditions.

### 2.6. Plasma Clearance

**Figure 6:**
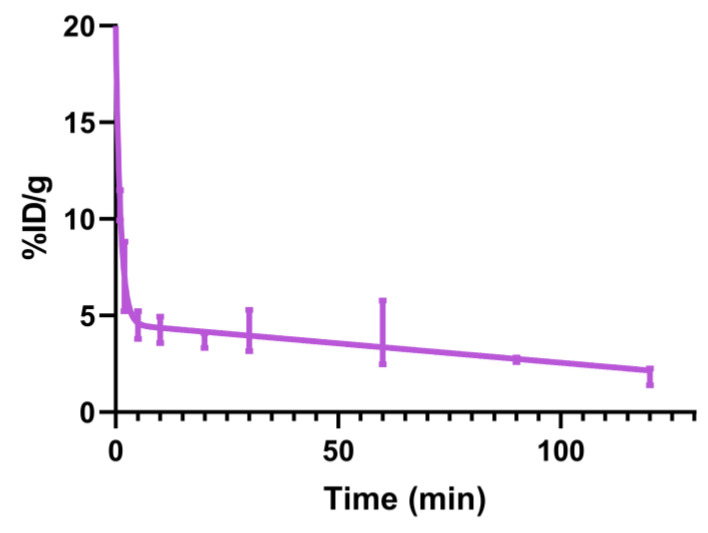
Activity curve of blood drug concentration (*n* = 3).

The plasma clearance experiment involved the collection of the blood drug concentration (%ID/g) at specific time points and fitting a curve to the data. The distribution-phase half-life (t_1/2α_) value of [^18^F]AlF-H_3_RESCA-FAPI was 0.76 min, and its clear-phase half-life (t_1/2β_) value was more than 60 min (Figure 6 and Table S5). The plasma clearance data revealed a rapid distribution phase and a longer clearance phase, suggesting that the tracer is rapidly taken up by tissues and slowly cleared from the body.

### 2.7. PET/CT Imaging

**Figure 7:**
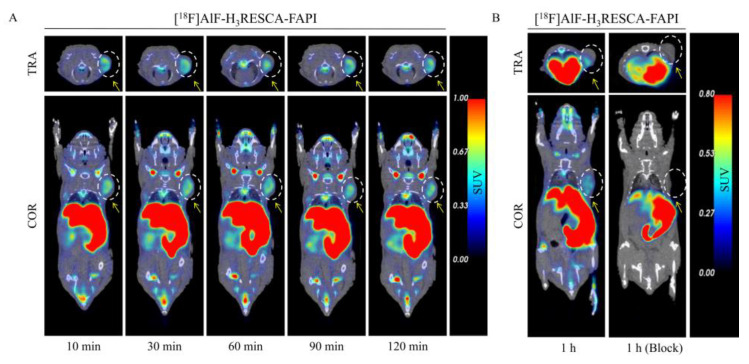
(**A**) Representative static PET images of [^18^F]AlF-H_3_RESCA-FAPI in U87MG xenograft models at different time points. (**B**) Representative static PET images of [^18^F]AlF-H_3_RESCA-FAPI in U87MG xenograft models with simultaneous injection of unlabeled DOTA-FAPI-04 as a competitor at 60 min. The yellow arrows point to the tumor.

Static PET imaging studies were performed in U87MG tumor-bearing nude mice to investigate the pharmacokinetics of [^18^F]AlF-H_3_RESCA-FAPI. The coronal and axial images of [^18^F]AlF-H_3_RESCA-FAPI at different scanning times are shown in Figure 7A. The tissue accumulation of the tracer is described by the SUV scale. With regard to tumors, [^18^F]AlF-H_3_RESCA-FAPI accumulated rapidly in U87MG tumor xenografts. A slow increase in tumor uptake was observed from 10 to 120 min (SUV_max_, from 0.70 ± 0.02 to 0.72 ± 0.06). This observation indicates that [^18^F]AlF-H_3_RESCA-FAPI exhibits a considerable retention time within the tumor.

However, the liver and intestine exhibited significant uptake values, possibly due to the certain lipid solubility of the tracer. Both the ADMET prediction and LogD_7.4_ measurement support this and predict increased intestinal absorption. In U87MG tumor model mice, from 10 to 120 min, [^18^F]AlF-H_3_RESCA-FAPI demonstrated rapid clearance kinetics in muscle (SUV_max_, from 0.029 ± 0.002 to 0.014 ± 0.002), intestine (SUV_max_, from 139.092 ± 43.447 to 53.872 ± 27.220), and liver (SUV_max_, from 2.251 ± 0.539 to 1.403 ± 0.370), while clearance from the kidney was comparatively slower. The relatively low uptake of [^18^F]AlF-H_3_RESCA-FAPI in the kidney resulted in less noticeable numerical changes (SUV_max_, less than 0.10), whereas the significantly high uptake in the intestine (SUV_max_, more than 15.60) led to more pronounced numerical changes. It requires further validation to determine whether the observed uptake value in the intestine is attributable to direct absorption within the intestinal tissue or to metabolites generated by the radioactive tracer that subsequently enter the intestine through metabolic pathways. The results of the blocking experiment, depicted in Figure 7B, demonstrate a successful reduction in tumor uptake following the co-administration of unlabeled DOTA-FAPI-04 (SUV_max_, unblocking vs. blocking at 1 h, 0.49 ± 0.18 vs. 0.04 ± 0.02, *p* = 0.0124). Moreover, significant reductions in uptake values were observed in the liver and bone.

### 2.8. Biodistribution

**Figure 8:**
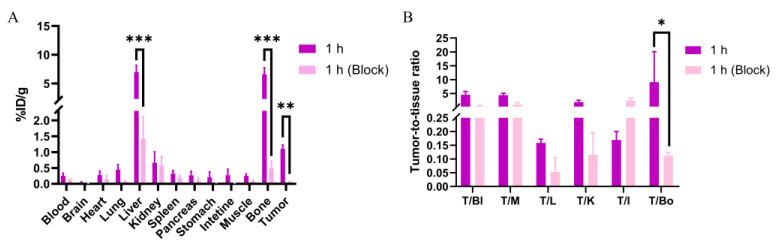
The ex vivo biodistribution of [^18^F]AlF-H_3_RESCA-FAPI in U87MG model mice 1 h after injection, with and without unlabeled DOTA-FAPI-04 as a blocking agent (**A**). The uptake ratio of [^18^F]AlF-H_3_RESCA-FAPI in tumor and blood, muscle, liver, kidney, intestine, and bone when co-administered with and without unlabeled DOTA-FAPI-04 as a blocking agent (**B**). All the data are expressed as mean ± SD values, *n* = 3 (* indicates *p* < 0.05, ** indicates *p* < 0.01, *** indicates *p* < 0.001).

Further biodistribution experiments of tumor-bearing mice were performed to validate the findings from PET imaging. The results indicated a significant uptake of [^18^F]AlF-H_3_RESCA-FAPI in the liver, where it is primarily metabolized. In comparison to the image results obtained from PET imaging, the primary distinction lies in the reduced intestinal uptake (Figure 8 and Table 2). This reduction can be attributed to the elimination of metabolites from the organs during the collection process, thereby demonstrating the rapid metabolism of [^18^F]AlF-H_3_RESCA-FAPI in the liver and intestines. Apart from this difference, the imaging results are largely consistent with those of PET.

The uptake of [^18^F]AlF-H_3_RESCA-FAPI in the tumor was 1.10 ± 0.12 %ID/g, which decreased to 0.05 ± 0.02 %ID/g after blocking, indicating a high level of specific uptake in the tumor (*p* = 0.004). Additionally, the tracer demonstrated significant tumor–muscle and tumor–blood ratios of 4.40 ± 0.71 and 4.56 ± 1.18, respectively (Figure 8 and Table S6). The substantial bone uptake of most ^18^F-labeled tracers remains evident, with the uptake quantified at 6.60 ± 1.16 %ID/g, which decreased to 0.49 ± 0.23 %ID/g after blocking (*p* < 0.001). Notwithstanding this characteristic, [^18^F]AlF-H_3_RESCA-FAPI demonstrates considerable promise as a PET tracer specifically targeting FAP. It is worth noting that, in the blocking experiments, a significant decrease in uptake was observed in both the liver and bone. This indicates that the uptake of [^18^F]AlF-H_3_RESCA-FAPI in these organs is not solely attributed to non-specific absorption or metabolic processes but also involves specific uptake. These tissues also reportedly exhibited FAP expression in previous studies [20].

### 2.9. Immunohistochemical

**Figure 9:**
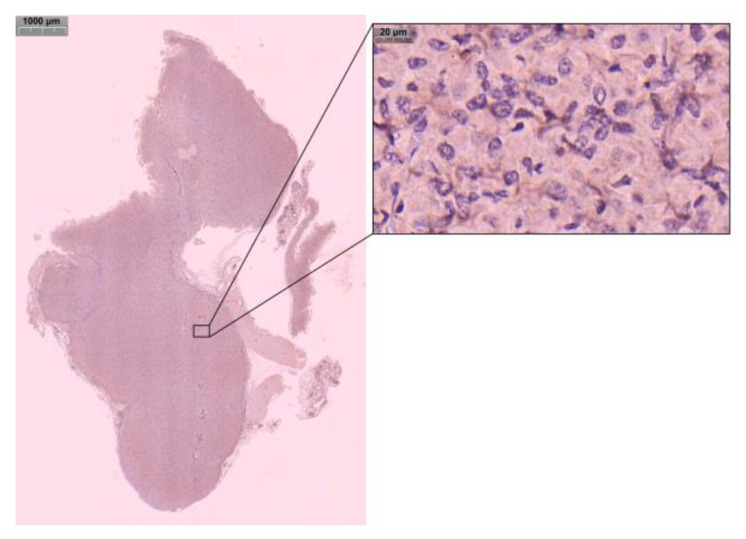
Immunohistochemical staining of FAP expression in U87MG tumor tissues.

The tumor of U87MG model mice was cut and soaked in formalin solution, and the staining pattern of FAP was obtained after the immunohistochemical staining experiment, which confirmed that all animal experiments of this tracer were carried out in animal models with the FAP expression (Figure 9).

## 3. Discussion

The present study introduces [^18^F]AlF-H_3_RESCA-FAPI, a novel radiotracer for PET imaging of tumors expressing FAP. The development of this tracer is significant as FAP is overexpressed in numerous epithelial carcinomas, making it an attractive target for both diagnostic and therapeutic applications. The results of our study provide a comprehensive evaluation of [^18^F]AlF-H_3_RESCA-FAPI, from its synthesis and radiolabeling to its in vitro and in vivo performance.

The molecular docking simulations and ADMET profiling study established the structural feasibility of H_3_RESCA-FAPI, predicting its interactions with FAP and its pharmacokinetic properties. The high binding affinity of H_3_RESCA-FAPI to FAP, as determined by SPR, is consistent with the docking results and underscores the potential of this tracer for the specific targeting of FAP-expressing tumors.

The radiochemical synthesis of [^18^F]AlF-H_3_RESCA-FAPI yielded a product with high RCP and RCYs, which is crucial for the practical application of the tracer in a clinical setting. The optimal conditions for radiolabeling, including pH, the molar ratio of aluminum chloride to the precursor, and the mass of H_3_RESCA-FAPI, were identified, providing a reliable protocol for the production of the tracer. Considering that the pH value of colloidal Al(OH)_3_ transformed from AlCl_3_ is above 5.5 [24] and the labeling yield is unstable in the buffer with pH = 5.5, the buffer with pH = 5.0 is selected as the best condition. The ability of [^18^F]AlF-H_3_RESCA-FAPI to achieve high radiolabeling yields at room temperature is a critical advantage. It simplifies the synthesis process, reducing the need for complex and costly equipment typically required for heating reactions. This not only lowers the barriers to production but also increases the feasibility of translating this tracer into a clinical setting. The high radiochemical yield of 92.4 ± 2.4% achieved under these mild conditions is particularly impressive and suggests that [^18^F]AlF-H_3_RESCA-FAPI is a robust candidate for PET imaging.

The octanol–water partition coefficient and stability assays demonstrated that [^18^F]AlF-H_3_RESCA-FAPI has reduced hydrophilicity compared to [^68^Ga]Ga-DOTA-FAPI-04, which may influence its biodistribution and pharmacokinetics. The plasma clearance data revealed a rapid distribution phase and a longer clearance phase, suggesting that the tracer is rapidly taken up by tissues and slowly cleared from the body.

PET/CT imaging and biodistribution studies in U87MG tumor-bearing mice showed specific and significant uptake of [^18^F]AlF-H_3_RESCA-FAPI in tumors, with high tumor-to-background ratios, indicating its potential for accurate tumor imaging. The high bone uptake observed is a common characteristic of ^18^F-labeled tracers [25,26,27,28,29,30,31,32,33,34,35]. The significant reduction in tumor uptake following the co-administration of unlabeled DOTA-FAPI-04 further confirms the specificity of [^18^F]AlF-H_3_RESCA-FAPI for FAP-expressing tumors. In addition, both PET/CT imaging and ex vivo biodistribution results showed that [^18^F]AlF-H_3_RESCA-FAPI exhibited a higher tumor uptake and tumor-to-normal tissue ratios compared to [^18^F]AlF-NOTA-FAPI-42, except in the liver and intestines (Figures S4 and S5).

While the results are promising, there are limitations to consider. The study’s findings are based on preclinical data, and further validation in clinical settings is necessary to confirm the tracer’s performance in humans. Additionally, the high uptake in the liver and intestine, although possibly due to the tracer’s lipid solubility, requires further investigation to understand its implications for diagnostic imaging.

In comparison to existing FAPI-based tracers, [^18^F]AlF-H_3_RESCA-FAPI demonstrates an improved labeling method, which is a significant advancement. However, a direct comparison with other ^18^F-labeled FAPI tracers in terms of tumor uptake and clearance rates is needed to fully assess its potential advantages. PET imaging studies have revealed that [^18^F]AlF-H_3_RESCA-FAPI exhibits a pronounced accumulation in the liver and intestine, potentially compromising the clarity of tumor imaging within these organs. Consequently, additional structural refinements may be warranted to achieve more favorable pharmacokinetic properties.

In conclusion, [^18^F]AlF-H_3_RESCA-FAPI shows considerable promise as a PET tracer for imaging of FAP-expressing tumors. Its high specificity, favorable pharmacokinetics, and robust stability make it a potential candidate for clinical translation, offering a new tool for non-invasive tumor imaging in future studies. However, further research is needed to address the limitations identified and to fully realize the potential of this tracer in clinical settings.

## 4. Materials and Methods

### 4.1. General

Except as indicated, compound **1** and compound **2** were procured from TanzhenBio (Nanchang, China), and other chemicals and reagents were procured from Merck (Shanghai, China) and used without additional purification. Characterization of synthesized compounds was performed using LC-MS on an Infinity Lab mass spectrometer equipped with an Agilent 1260 series HPLC system (Agilent Technologies, Santa Clara, CA, USA) and an Extend-C18 column (50 mm × 2.1 mm, 1.8 μm), monitored at a UV wavelength of 254 nm. Radioisotope ^18^F^−^ was produced in a medical cyclotron (HM-10, Sumitomo Heavy Industries Ltd., Tokyo, Japan) via the ^18^O (p, n) ^18^F reaction. Quality control of the radiotracers was conducted using HPLC with a C18 column (C18-TH, 5 μm, 100 Å, 150 mm × 4.6 mm, Morhchem Technologies Inc., City of Industry, CA, USA) and a 1260 Quat pump VL, a 1260 DAD VL detector, a 1260 Vialsampler, and a γ-detector for radioactivity measurement (Eckert and Ziegler, Berlin, Germany). The mobile phase composition of water and acetonitrile was adjusted according to the specific compounds. Radioactivity measurements were taken with a CRC-55T activity meter (The China National Nuclear Corporation, Beijing, China).

### 4.2. Molecular Docking

Three-dimensional structures of DOTA-FAPI-04, NOTA-FAPI-42, and H_3_RESCA-FAPI were constructed using Chem3D 20.0 (PerkinElmer, Shelton, CT, USA). The resultant mol2 files were converted to .pdb format with PyMol 2.6.0. The target protein (PDB ID: 1Z68) underwent preparation for docking by adding hydrogen atoms with AutoDock Tools 1.5.7 (The Scripps Research Institute, La Jolla, CA, USA). A docking grid, encompassing the catalytic site of FAPα, was defined. This grid was centered around the catalytic triad—Ser^624^, Asp^702^, and His^734^—using AutoGrid 4.0, which set the grid dimensions at 24 × 24 × 24 Å³ and a grid spacing of 1.000 Å, to ensure comprehensive coverage of the catalytic site. The coordinates for the grid box center were established at x = 25.071 Å, y = 10.676 Å, and z = 25.618 Å. Molecular docking, carried out with Autodock Vina 1.1.2, evaluated the influence of various chelator groups on the binding affinity of FAPI-4 to FAP. Each compound underwent three independent docking simulations. The lowest-energy conformation from each set of runs, as identified by Autodock Vina, was selected for visualization with PyMol 2.6.0.

### 4.3. ADMET Profiling Studies

The ADMET properties of FAPI-based ligands were predicted using the ADMETlab 3.0 web tools, which facilitate a comprehensive ADMET analysis. Ligand structures, described by their Simplified Molecular Input Line Entry System (SMILES) representations, were input into the platform to generate 2D structural files. This enabled ADMETlab 3.0 to compute and evaluate essential ADMET parameters effectively. Notably, the SMILES strings for two specific ligands were generated with ChemDraw 20.0 and are detailed in Supplementary Texts S1 and S2.

### 4.4. Chemistry

2,2′-(((1R,2R)-2-((carboxymethyl)(4-(2-(4-(3-((4-((2-((S)-2-cyano-4,4-difluoropyrrolidin-1-yl)-2-oxoethyl)carbamoyl)quinolin-6-yl)oxy)propyl)piperazin-1-yl)-2-oxoethyl)benzyl)amino)cyclohexyl)azanediyl)diacetic acid (**3**). Compound **1** (50 mg, 103 μmol) was dissolved in DMSO (4 mL), followed by the addition of compound **2** (77 mg, 131 μmol) and triethylamine (Et_3_N, 100 μL, 719 μmol). The mixture was stirred at room temperature (20 °C) for 4 h and then concentrated under reduced pressure. Purification was achieved using semi-preparative HPLC with a COSMOSIL 5C18-MS-II column (120 Å, 5 μm, 250 × 10.0 mm) and a gradient elution of acetonitrile/water (0.1 M TEAB) initiated at 16/84 (*v*/*v*) for 20 min at a flow rate of 2 mL/min. The solvent was removed under reduced pressure to yield compound **3** as a white powder (32.2 mg, 34.6%). LC-MS (ESI) analysis showed a molecular ion peak at *m*/*z* = 904.8 [M+H]^+^.

### 4.5. Binding Affinity Measurement

In order to determine the binding kinetics between ligands and human FAP protein (10464-H07H, Sino Biological Inc., Beijing, China), the Biacore SPR interaction study was conducted. The binding affinity is expressed by the equilibrium dissociation constant (K_D_) (M), which is calculated from the values of the measured binding rate constant (k_a_) (M^−1^ s^−1^) and dissociation rate (k_d_) (s^−1^).

### 4.6. Radiochemistry and Quality Control

^18^F^−^ was loaded onto an activated QMA ion exchange column (Waters GmbH, Eschborn, Germany), which had been previously eluted with 1 mL of saline and air-dried. The column containing ^18^F^−^ was subsequently eluted with 1 mL of saline to obtain a Na^18^F solution.

Reaction conditions were screened using five different pH buffers: a NaHP buffer at pH 6.0 (0.2 M) and a sodium acetate buffer ranging from pH 4 to 5.5 (0.2 M). For the reactions, 100 μL of Na^18^F solution (370 MBq) and 12 μL of AlCl_3_ solution (1.28 mM, prepared in 0.2 M buffer solution) were added to a reaction vial. The mixture was incubated at room temperature for 5 min. Subsequently, 300 μL of buffer solution at varying pH values and 200 μL of H_3_RESCA-FAPI solution (0.1 mg/mL, prepared in deionized water) were added, and the mixture was allowed to react for 15 min at room temperature. The radiolabeling yields across different pH conditions were quantified by HPLC.

Utilizing the pH buffer that yielded the highest radiolabeling efficiency, 10 μL of Na^18^F solution (37 MBq) and variable volumes of AlCl_3_ solution (either 12 or 12.4 μL, 1.07 mM, prepared in the selected buffer) were added to a reaction vial and incubated at room temperature for 5 min. This was followed by the addition of 200 μL of H_3_RESCA-FAPI solution (0.1 mg/mL, prepared in the selected buffer) and 300 μL of the same buffer. The reaction proceeded for 15 min at room temperature. Radiolabeling efficiencies were then calculated by HPLC, analyzing different AlCl_3_-to-ligand molar ratios (0.58 or 0.60).

In a subsequent experiment, the selected volume of AlCl_3_ was added to a reaction flask along with 100 μL of Na^18^F solution (185 MBq) and incubated at room temperature for 5 min. Different volumes of H_3_RESCA-FAPI solution (200, 500, or 1000 μL, 0.1 mg/mL, prepared in the selected buffer) were then introduced, maintaining the same volume with the selected buffer. The reaction was sustained for 15 min at room temperature. Radiolabeling yields for varying ligand masses (20, 50, or 100 μg) were determined by HPLC.

The purification protocol for the Sep-Pak Light C18 column involves first eluting the reaction solution with 5 mL of pure water through the activated column. Subsequently, the product is eluted into a sterile vacuum flask with 1 mL of a 50% ethanol solution, followed by the addition of 4 mL of saline, rendering the preparation ready for use. For HPLC analysis, the mobile phase consisted of an aqueous solution containing 0.10% trifluoroacetic acid (Phase A) and acetonitrile (Phase B). An isocratic elution was employed, with a mobile phase consisting of 18% Phase B, which was kept constant throughout the analysis. The flow rate was set at 1 mL/min. UV detection was performed at a wavelength of 254 nm.

### 4.7. Partition Coefficient

Radiotracers [^68^Ga]Ga-DOTA-FAPI-04 and [^18^F]AlF-H_3_RESCA-FAPI (0.37 MBq, about 50 µL) were added to tubes containing n-octanol/PBS mixture (1 mL, 1:1). The mixture was vortexed vigorously for 5 min at ambient temperature and then centrifuged for five min at 8000 rpm. In each phase, three samples (each 50 µL) were removed and measured in γ-counter (PerkinElmer). The partition coefficient was expressed as an equation as follows:

(1) $${\mathrm{LogD}}_{7.4}=\mathrm{Log}\frac{CPM(n-octanol)}{CPM(PBS)}$$

### 4.8. In Vitro and In Vivo Stability

[^18^F]AlF-H_3_RESCA-FAPI (10 MBq) in a mixture of ethanol and water was added to PBS (600 µL) or FBS (GE Healthcare, Chicago, IL, USA) (600 µL) and incubated at 37 °C for 2 h. And then, a sample of PBS or FBS (100 µL) was injected into the HPLC system for analysis. At 60 min p.i. of [^18^F]AlF-H_3_RESCA-FAPI (37 MBq/mouse), the mice were sacrificed. The urine was then collected and analyzed by HPLC directly.

### 4.9. Pharmacokinetics in Normal Mice

The distribution of [^18^F]AlF-H_3_RESCA-FAPI (0.93 MBq) in blood was assessed at 1, 2, 5, 10, 20, 30, 60, 90, and 120 min using normal mice. The mice (*n* = 3 in each group) had their blood drawn at the selected time. All the collected blood was quickly removed and weighed. The radio activity was counted using a γ-counter, and the results were expressed as %ID/g.

### 4.10. PET Imaging

U87MG tumor-bearing mice (*n* = 3 per group) underwent PET imaging using an IRIS micro-PET/CT scanner (Inviscan SAS, Strasbourg, France). Mice were anesthetized and put in prone position for the scans. Static PET images were acquired at 10, 30, 60, 90, and 120 min p.i. of [^18^F]AlF-H_3_RESCA-FAPI (4−6 MBq/mouse). For the blocking study, [^18^F]AlF-H_3_RESCA-FAPI was co-injected with unlabeled DOTA-FAPI-04 (0.5 mg/mouse), followed by a 1 h static PET scan. The PET data were reconstructed using a three-dimensional ordered-subset expectation maximization (3D-OSEM) algorithm, which incorporates a Monte Carlo simulation for accurate modeling of the detector response. The reconstructed images were analyzed using Avatar 1.2 software (Pingseng, Kunshan, China) for semi-quantitative assessment.

### 4.11. Biodistribution Studies

The biodistribution of [^18^F]AlF-H_3_RESCA-FAPI (4.32–5.45 MBq) was evaluated in athymic nude mice bearing U87MG xenografts at 1 h p.i. A subset of mice received a concurrent injection of the radiotracer and the competitor, DOTA-FAPI-04 (0.5 mg per mouse), to assess blocking effects. At 60 min p.i., the mice (*n* = 3 per group) were euthanized, and selected organs, tumors, and blood were harvested, weighed, and measured for radioactivity using a γ-counter. The results were expressed as %ID/g.

### 4.12. Immunohistochemical Staining

U87MG tumor tissues were harvested post-euthanasia and processed into paraffin-embedded sections for immunohistochemical analysis. Sections underwent deparaffinization in xylene and rehydration through graded ethanol. Antigen retrieval was performed, followed by overnight incubation with a rabbit polyclonal anti-FAP primary antibody (1:50, AF0739, Affinity, San Francisco, CA, USA) at 4 °C. After rinsing, sections were incubated with a goat anti-rabbit secondary antibody (PV-9000, ZSGB-BIO, Beijing, China) for 1 h at room temperature. FAP expression was visualized using diaminobenzidine (DAB), counterstained with hematoxylin, dehydrated, cleared, and mounted. Positive staining was assessed and photographed under a microscope.

### 4.13. Statistical Analysis

Quantitative data are expressed as mean ± standard deviation (SD). The statistical significance between 2 independent groups was determined by Student’s *t*-test. A *p*-value of less than 0.05 was considered statistically significant. All statistical analyses were performed using GraphPad Prism version 8.0.1 (Graph Software, La Jolla, CA, USA).

## 5. Conclusions

In this study, a novel radiopharmaceutical, [^18^F]AlF-H_3_RESCA-FAPI, was synthesized utilizing the established FAPI scaffold. In comparison to existing FAPI-based tracers, [^18^F]AlF-H_3_RESCA-FAPI demonstrates an improved labeling method, which is a significant advancement. It was evaluated in vitro and in vivo and showed the specific uptake of FAP-expressing tumors in mice. It exhibited an extremely mild labeling process and high specificity. Therefore, this novel FAP-targeted radioactive tracer may be a promising tracer for non-invasive tumor imaging in subsequent clinical research, but its structure needs to be further modified to obtain better pharmacokinetic characteristics.

## Figures and Tables

**Table 1:** Affinity score of the optimal conformation determined by molecular coupling (AutoDock Vina 1.1.2), as well as the RMSD values.

Ligand	Affinity (kcal/mol)	rmsd 1.b./rmsd u.b.
DOTA-FAPI-04	−9.1	0.000
NOTA-FAPI-42	−8.9	0.000
H_3_RESCA-FAPI	−10.0	0.000

**Table 2:** Biodistribution data of [^18^F]AlF-H_3_RESCA-FAPI in U87MG model mice 1 h after injection, with and without unlabeled DOTA-FAPI-04 as a blocking agent, *n* = 3 (%ID/g).

Tissue	60 min	60 min Blocking
Blood	0.25 ± 0.08	0.20 ± 0.04
Brain	0.03 ± 0.03	0.01 ± 0.00
Heart	0.27 ± 0.13	0.14 ± 0.12
Lung	0.44 ± 0.16	0.06 ± 0.03
Liver	6.99 ± 1.18	1.43 ± 0.70
Kidney	0.66 ± 0.34	0.58 ± 0.29
Spleen	0.32 ± 0.10	0.20 ± 0.07
Pancreas	0.26 ± 0.13	0.09 ± 0.08
Stomach	0.20 ± 0.17	0.02 ± 0.01
Intestine	0.27 ± 0.19	0.02 ± 0.00
Muscle	0.26 ± 0.06	0.07 ± 0.05
Bone	6.60 ± 1.16	0.49 ± 0.23
Tumor	1.10 ± 0.12	0.05 ± 0.02

## Data Availability

Datasets generated during and analyzed during the current study are available from the corresponding author upon reasonable request.

## Supplementary Materials

The following supporting information can be downloaded at: https://www.mdpi.com/article/10.3390/ph18020277/s1, Table S1: Predicted Physico-chemical, Druglikeness and Molecular properties of ligands, Table S2: Predicted values of pharmacokinetics, ADMET properties and drug similarity of DOTA-FAPI-04, Table S3: Predicted values of pharmacokinetics, ADMET properties and drug similarity of H_3_RESCA-FAPI, Table S4: Data of Radiolabling yield, Table S5: The blood drug concentration of [^18^F]AlF-H_3_RESCA-FAPI, Table S6: Tumor to tissue ratio of [^18^F]AlF-H_3_RESCA-FAPI in U87MG model mice, Figure S1: LC-MS spectrometry for H_3_RESCA-FAPI, Figure S2: HPLC spectrometry for [^19^F]AlF-H_3_RESCA-FAPI, Figure S3: Quality control of [^18^F]AlF-H_3_RESCA-FAPI, Figure S4: PET images of [^18^F]AlF-NOTA-FAPI-42 in U87MG models, Figure S5: The ex vivo Biodistribution of [^18^F]AlF-NOTA-FAPI-42 in U87MG models, Text S1: The SMILES format of DOTA-FAPI-04, Text S2: The SMILES format of H_3_RESCA-FAPI.

## Abbreviations

The following abbreviations are used in this manuscript:

HPLC	High-performance liquid chromatography
RCY	Radiochemical yield
RCP	Radiochemical purity
PET	Positron emission tomography
NOTA	Hexadentate ligand 1,4,7-triazacyclononane-1,4,7-triacetic acid
RESCA	Restrained complexing agent
DOTA	2,2′2″,2-(1,4,7,10-Tetraazacyclododecane-1,4,7,10-tetrayl)tetraacetic acid
QMA	Quaternary methyl ammonium
SUV	Standardized uptake value
NODAGA	N,N′,N″-(1,4,7-triazacyclononane-1,4,7-triyl)triacetic acid
2-AMPTA	2-Aminomethyl-4,5-dihydroxyphenylacetic acid
DTPA	Diethylenetriaminepentaacetic acid
NaHP	Na2HPO4
Am	Molar activity
Bq	Becquerel
FAP	Fibroblast activation protein
CAFs	Cancer-associated fibroblasts
%ID/g	Percentage of injected dose per gram of tissue
SPR	Surface plasmon resonance
p.i.	Post-injection
