# Supplementary material for: Design, Synthesis, and Biological Evaluation of a Novel [18F]AlF-H3RESCA-FAPI Radiotracer Targeting Fibroblast Activation Protein
Source: Pharmaceuticals (Basel). 2025 Feb 19;18(2):277. doi: 10.3390/ph18020277 (PMC11859916; doi:10.3390/ph18020277)
Supplement: Supplementary file 1 [file pharmaceuticals-18-00277-s001.zip › pharmaceuticals-3444241-supplementary.pdf]

Supplementary Materials

Design, Synthesis and Biological Evaluation of a Novel [18F]AlF-H_3RESCA-FAPI Radiotracer Targeting Fibroblast Activation Protein.

Table S1. Predicted Physico-chemical, Druglikeness and Molecular properties of ligands:

S/N	Parameter	DOTA-FAPI-04	H_3RESCA-FAPI
1	LogP	-1.982	-0.639
2	TPSA	243.73	237.25
3	natoms	116	119
4	MW	872.4	904.39
5	nON(HBA*)	20.0	18.0
6	nOHNH(HBD**)	4.0	4.0
7	Lipinski Ro5	Rejected	Rejected
	Pfizer	Accepted	Accepted
	GSK	Rejected	Rejected
	Golden Triangle	Rejected	Rejected
8	nrotb	19.0	23.0
9	volum	833.345	881.365

Note: *HBA: Hydrogen Bond Acceptors (as total number of nitrogen and oxygen atoms), **HBD: Hydrogen Bond Donors (as total number of oxygen−hydrogen and nitrogen−hydrogen bonds)

Table S2. Predicted values of pharmacokinetics, ADMET properties and drug similarity of DOTA-FAPI-04:

S/N	Absorption		Distribution		Metabolism		Excretion and Toxicity	
1	Water Solubility (Log S)	-3.767	Volume distribution (VD) of a drug in blood plasmas	-0.023	CYP2D6 inhibitor	---	Total drug clearance log (CLtot)	1.033
2	Lipid Solubility (Log P)	-1.982			CYP3A4 inhibitor	---	AMES toxicity	0.03
3	Caco-2 Permeabilit y	-5.371	Plasma protein binding (PPB)	38.372	CYP1A2 inhibitor	---	hERG Blockers	0.999
4	MDCK permeability	-4.994	BBB permeability	---	CYP2C19 inhibitor	---		
5	Human intestinal absorption (HIA)	+++	The fraction unbound in blood plasmas (Fu)	59.522	CYP2C9 inhibitor	---		
6	QED value (a measure of drug likeness) Attractive> 0.67	0.15						

Note: For the classification endpoints , the prediction probability values are transformed into six symbols: 0-0.1 (---), 0.1-0.3 (--), 0.3-0.5 (-), 0.5-0.7 (+), 0.7-0.9 (++), and 0.9-1.0 (+++).

Table S3. Predicted values of pharmacokinetics, ADMET properties and drug similarity of H_3RESCA-FAPI:

S/N	Absorption		Distribution		Metabolism		Excretion and Toxicity	
1	Water Solubility (Log S)	-2.327	Volume distribution (VD) of a drug in blood plasmas	-0.096	CYP2D6 inhibitor	---	Total drug clearance log (CLtot)	1.185
2	Lipid Solubility (Log P)	-0.639			CYP3A4 inhibitor	---	AMES toxicity	0.08
3	Caco-2 Permeabilit y	-5.626	Plasma protein binding (PPB)	68.838	CYP1A2 inhibitor	---	hERG Blockers	0.983
4	MDCK permeability	-4.994	BBB permeability	---	CYP2C19 inhibitor	---		
5	Human intestinal absorption (HIA)	--	The fraction unbound in blood plasmas (Fu)	27.905	CYP2C9 inhibitor	---		
6	QED value (a measure of drug likeness) Attractive> 0.67	0.119						

Note: For the classification endpoints , the prediction probability values are transformed into six symbols: 0-0.1 (---), 0.1-0.3 (--), 0.3-0.5 (-), 0.5-0.7 (+), 0.7-0.9 (++), and 0.9-1.0 (+++).

O=C(N1CCN(CCCOC2=CC=C3N=CC=C(C(NCC(N4[C@H](C#N)CC(F)(F)C4)=O)=O)C3=C2)CC1)CN5CCN(CC(O)=O)CCN(CC(O)=O)CCN(CC(O)=O)CC5

Text S1. The SMILES format of DOTA-FAPI-04.

O=C(C1=CC=NC2=CC=C(OCCCN3CCN(C(CC4=CC=C(CN([C@@H]5CCCC[C@H]5N(CC(O)=O)CC(O)=O)CC(O)=O)C=C4)=O)CC3)C=C12)NCC(N6[C@H](C#N)CC(F)(F)C6)=O

Text S2. The SMILES format of H_3RESCA-FAPI.

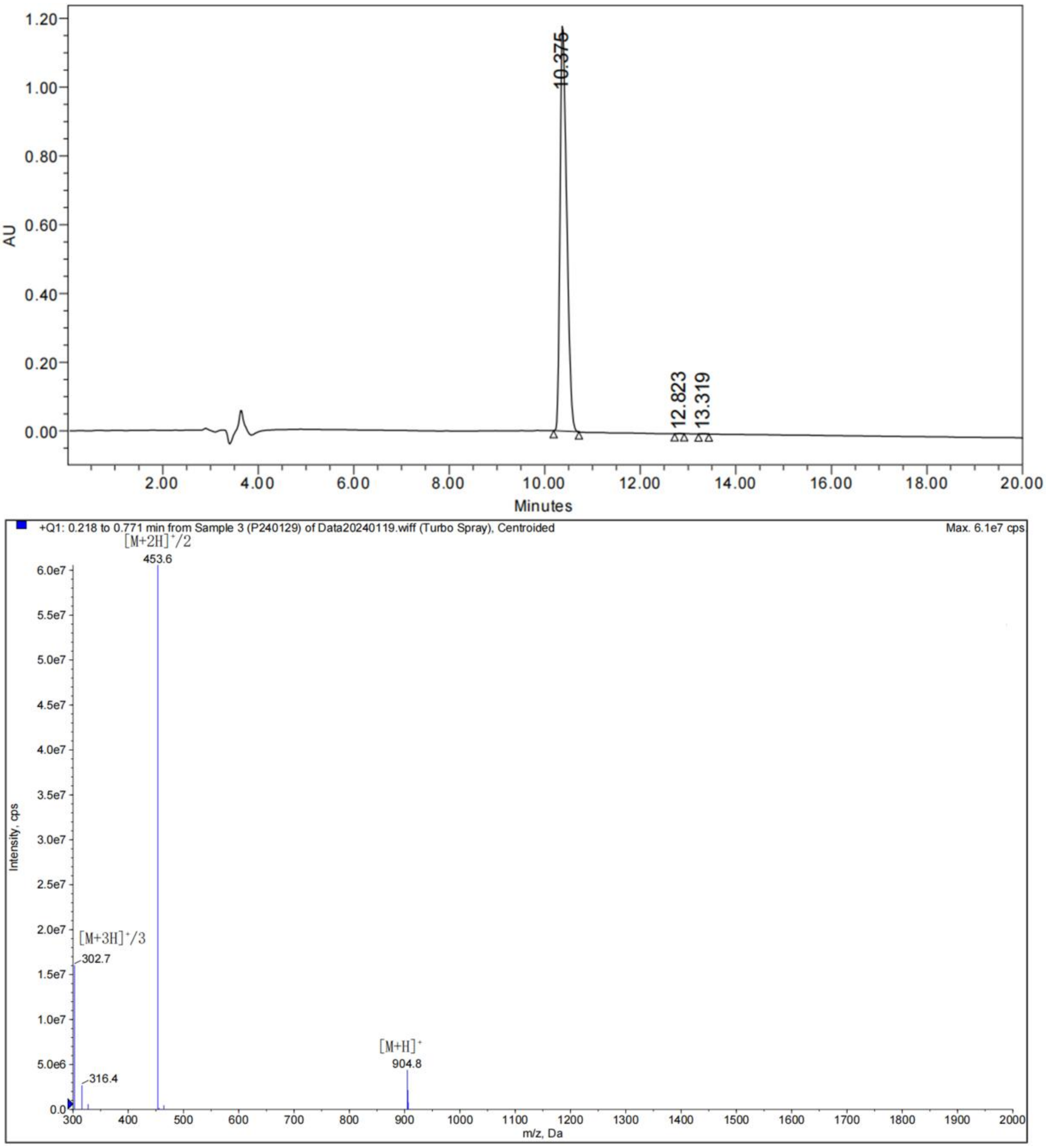

Figure S1. LC-MS spectrometry for H_3RESCA-FAPI.

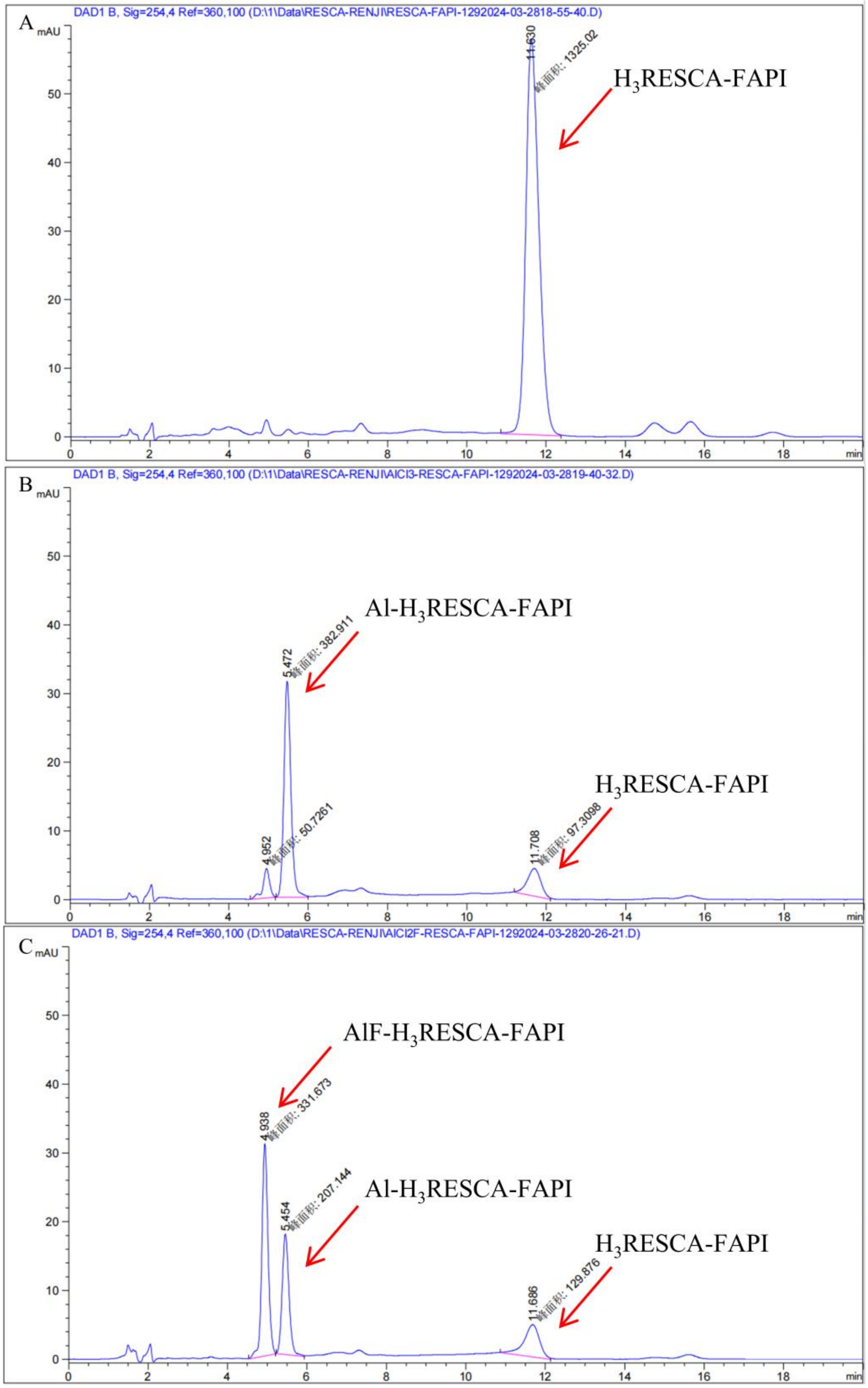

Figure S2. HPLC spectrometry for H_3RESCA-FAPI (A) and reaction solution for chelating reaction with $AlCl_3$ (B) and continue to add that reaction solution of $K^{19}F$ for fluorination reaction (C). HPLC condition serve as the method for quality control.

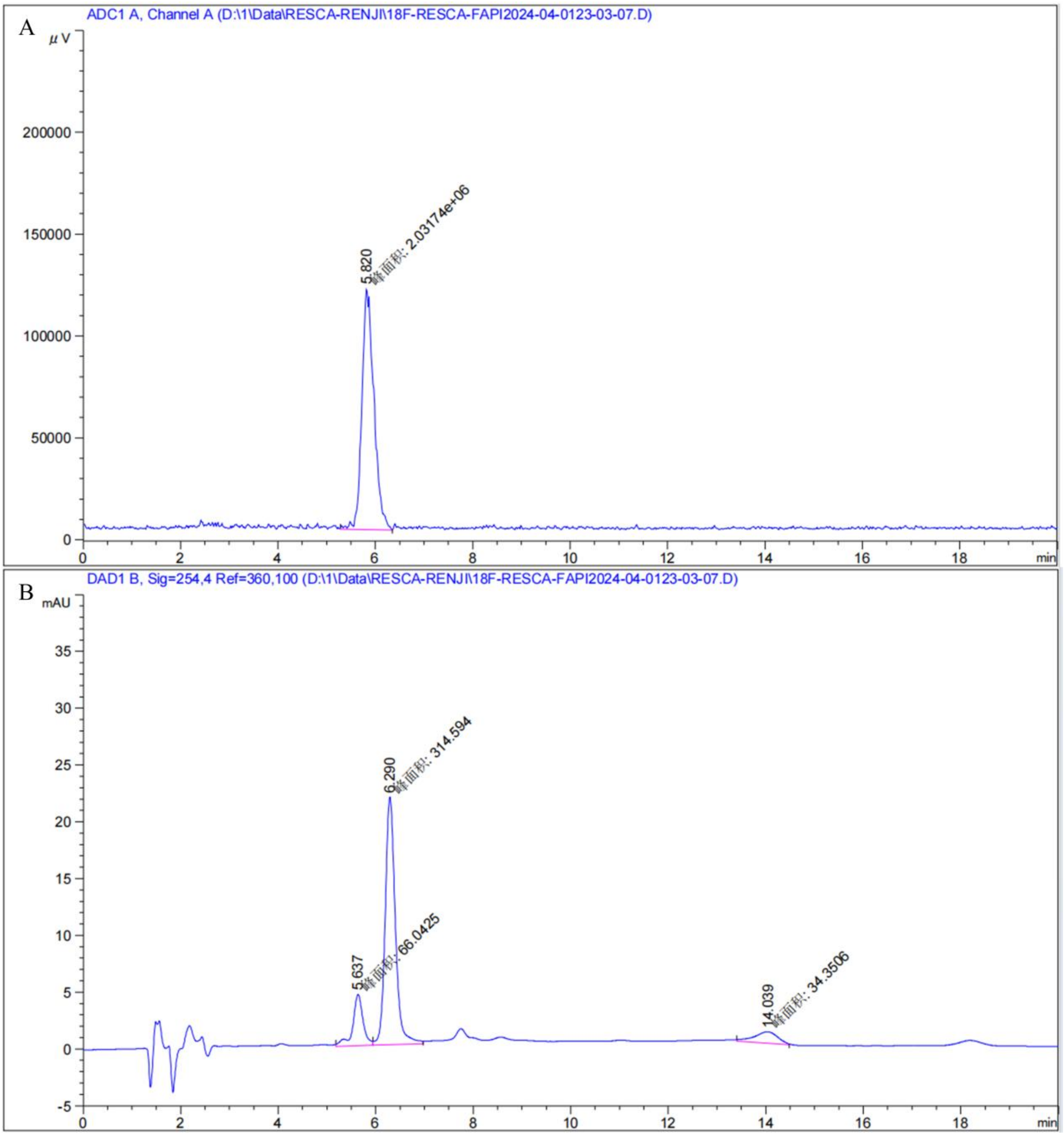

Figure S3. Radio-HPLC spectrometry for [^{18}F]AlF-H_3RESCA-FAPI (A) and HPLC spectrometry for [^{18}F]AlF-H_3RESCA-FAPI at a wavelength of 254 nm (B).

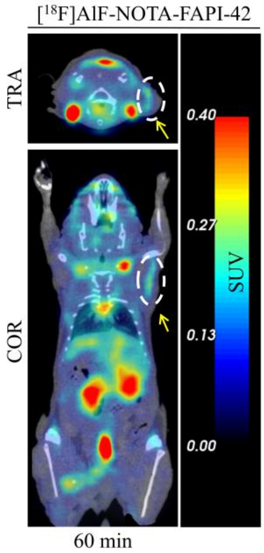

Figure S4. Representative static PET images of [18F]AlF-NOTA-FAPI-42 in U87MG xenograft models at 60 min p.i.. The yellow arrows point to the tumor.

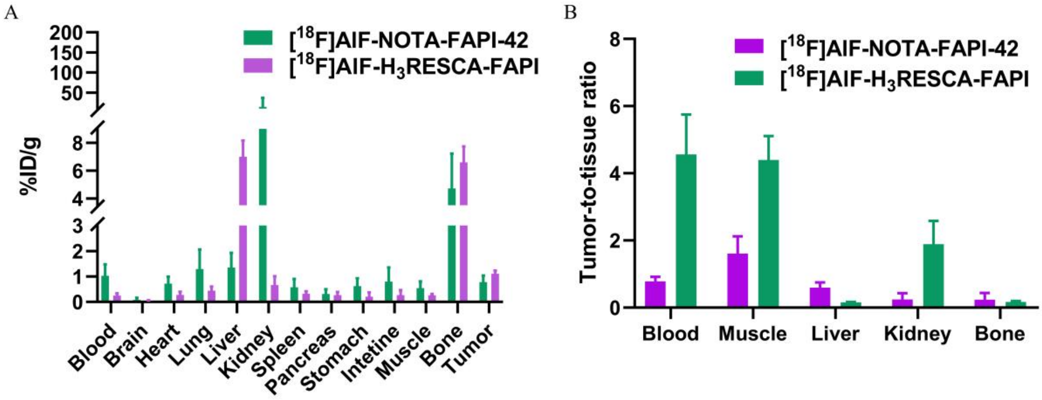

Figure S5. The *ex vivo* Biodistribution of [18F]AlF-H_3RESCA-FAPI and [18F]AlF-NOTA-FAPI-42 in U87MG model mice at 1 h p.i. (A) and the uptake ratio of both tracers in tumor, blood, muscle, liver, kidney and bone (B). All the data are expressed as mean ± SD values, *n = 3*.

Table S4. Radiolabling yield at real-time temperature and under different reaction conditions (including pH of buffer, $AlCl_3$ and precursor dosage).

t (°C)	pH	$n_{(AlCl3)}$:$n_{(precursor)}$	$m_{(precursor)}$ (μg)	Radiolabling yield (%)
15	4.0	0.58	20	22.38
15	4.5	0.58	20	48.81
15	5.0	0.58	20	91.88
15	5.5	0.58	20	97.63
15	6.0	0.58	20	0.00
28	5.0	0.58	20	94.56
28	5.0	0.60	20	81.82
10	5.0	0.58	20	72.32
10	5.0	0.58	50	96.68
10	5.0	0.58	100	85.87

Table S5. The blood drug concentration (%ID/g) in normal mice at 1 min, 2 min, 5 min, 10 min, 20 min, 30 min, 60 min, 90 min and 120 min (*n=3*).

time (min)	%ID/g
1	10.71 ± 0.70
2	7.14 ± 1.68
5	4.61 ± 0.66
10	4.31 ± 0.69
20	3.74 ± 0.36
30	4.27 ± 0.90
60	4.16 ± 1.45
90	2.77 ± 0.10
120	1.86 ± 0.37

Table S6. Tumor to tissue ratio of [^{18}F]AlF-H_3RESCA-FAPI in U87MG model mice 1 h after injection, with and without unlabeled DOTA-FAPI-04 as a blocking agent (*n = 3*).

	Tumor to tissue ratio	
	60 min unblocking	60 min blocking
Blood	4.56 ± 1.18	0.50 ± 0.27
Muscle	4.40 ± 0.71	1.01 ± 0.67
Liver	0.16 ± 0.01	0.05 ± 0.05
Kidney	1.89 ± 0.69	0.12 ± 0.08
Bone	0.17 ± 0.03	0.11 ± 0.01
Intetine	9.10 ± 10.97	2.49 ± 0.91
